# Room tilt illusion and subclavian steal – a case report

**DOI:** 10.1186/s12883-020-01947-2

**Published:** 2020-10-08

**Authors:** Kathrine Arntzen, Karl B. Alstadhaug

**Affiliations:** 1 Department of Neurology, Nordland Hospital, 8092 Bodø, Norway; 2 Institute of Clinical Medicine, The Arctic University of Tromsø, Tromsø, Norway

**Keywords:** Room tilt illusion, Inverted vision, Subclavian steal, Stroke, Case report

## Abstract

**Background:**

Room tilt illusion (RTI) is a rare symptom of higher vestibular dysfunction, consisting of a transient vertical rotation of the visual scene in the sagittal or coronal plane, most often 90^o^ or 180^o^, without any alteration in shape, size and color of objects.

**Case presentation:**

A 63-year-old woman with a history of hypertension and chronic obstructive pulmonary disease went through an uncomplicated aortobifemoral graft surgery due to aortoiliac occlusive disease. Post-operatively she experienced five episodes, lasting from 10 to 30 min, with RTI; 90^o^ forward rotation of the visual scene in the sagittal plane. Work-up revealed subclavian steal grade 3, and transient ischemia of the central vestibular system of the brainstem was the presumed mechanism.

**Conclusion:**

The course of episodic RTIs is often benign, but RTI may represent ischemia in the posterior cerebral circulation. Both stroke and otoneurologic workup are recommended. To our knowledge, this is the first case of RTI associated with subclavian steal reported.

## Background

The phenomenon of room tilt illusion (RTI) was first described in a patient thought to suffer from hysteria in 1805 [1]. Relatively few cases have since been reported. In 2012 Sierra-Hidalgo and colleagues presented 13 own cases, and reviewed 135 more that had been previously published [2]. RTI is the perception that the visual scene is transiently tilted 90^o^ or rotated 180^o^ (upside-down), and represents a disorder of higher vestibular functions [3], a transient mismatch between the cortical visual and the vestibular three-dimensional (3D) coordinate maps [4]. The underlying cause of RTI varies, from peripheral vestibular [3] and neurological disorders [5] to migraine [6] and stroke [7]. We report a patient with RTI presumed to represent a manifestation of her subclavian steal.

## Case presentation

**Fig. 1:**
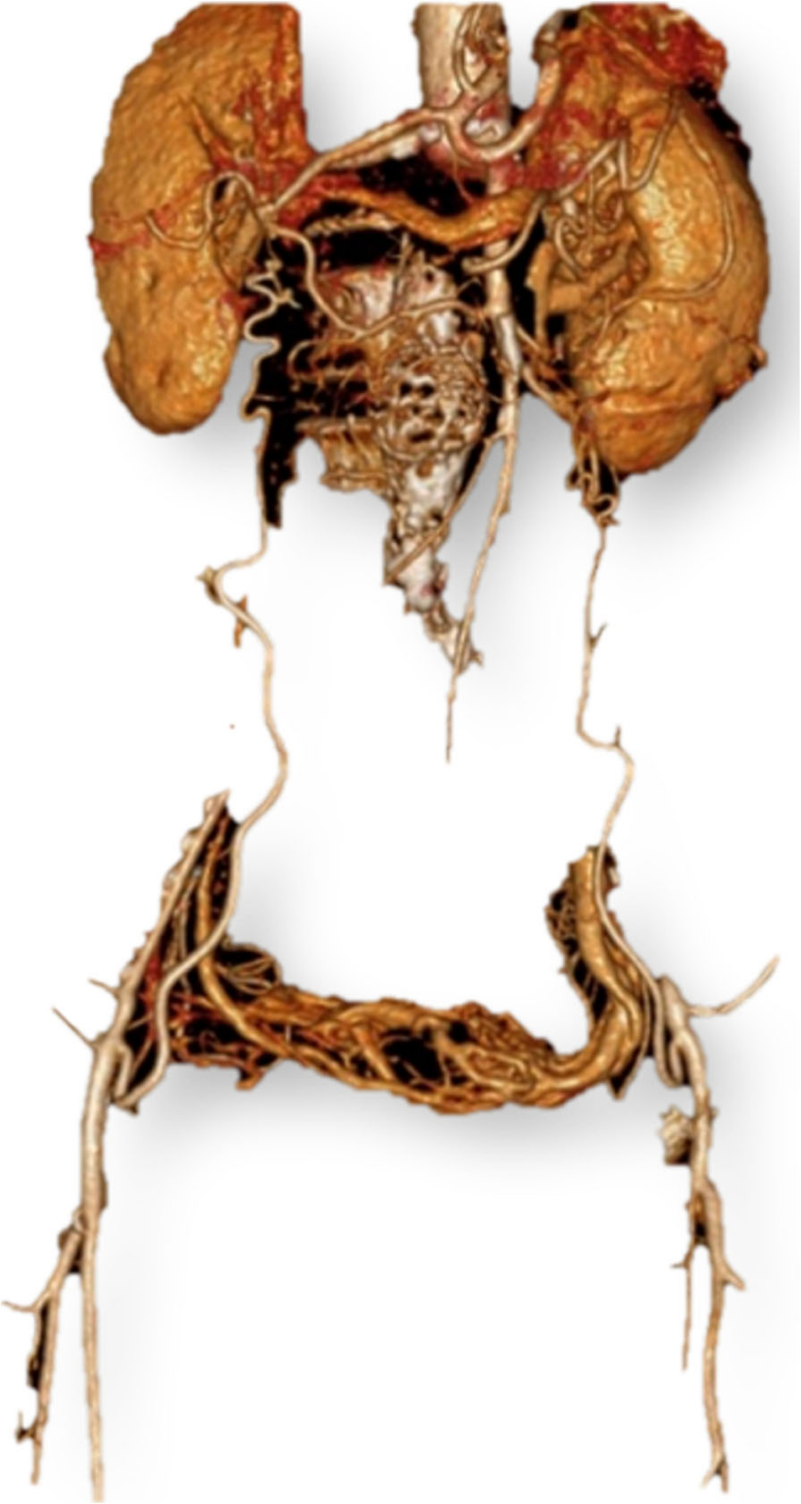
Three-dimensional digital subtraction CT-angiography demonstrating the patient’s abdominal aortic occlusion with collateral blood supply to the legs. (The basic images were obtained with a Philips Ingenuity 128 CT-scanner, and the 3D reconstruction was made by using IntelliSpace Portal 8.0)

**Fig. 2:**
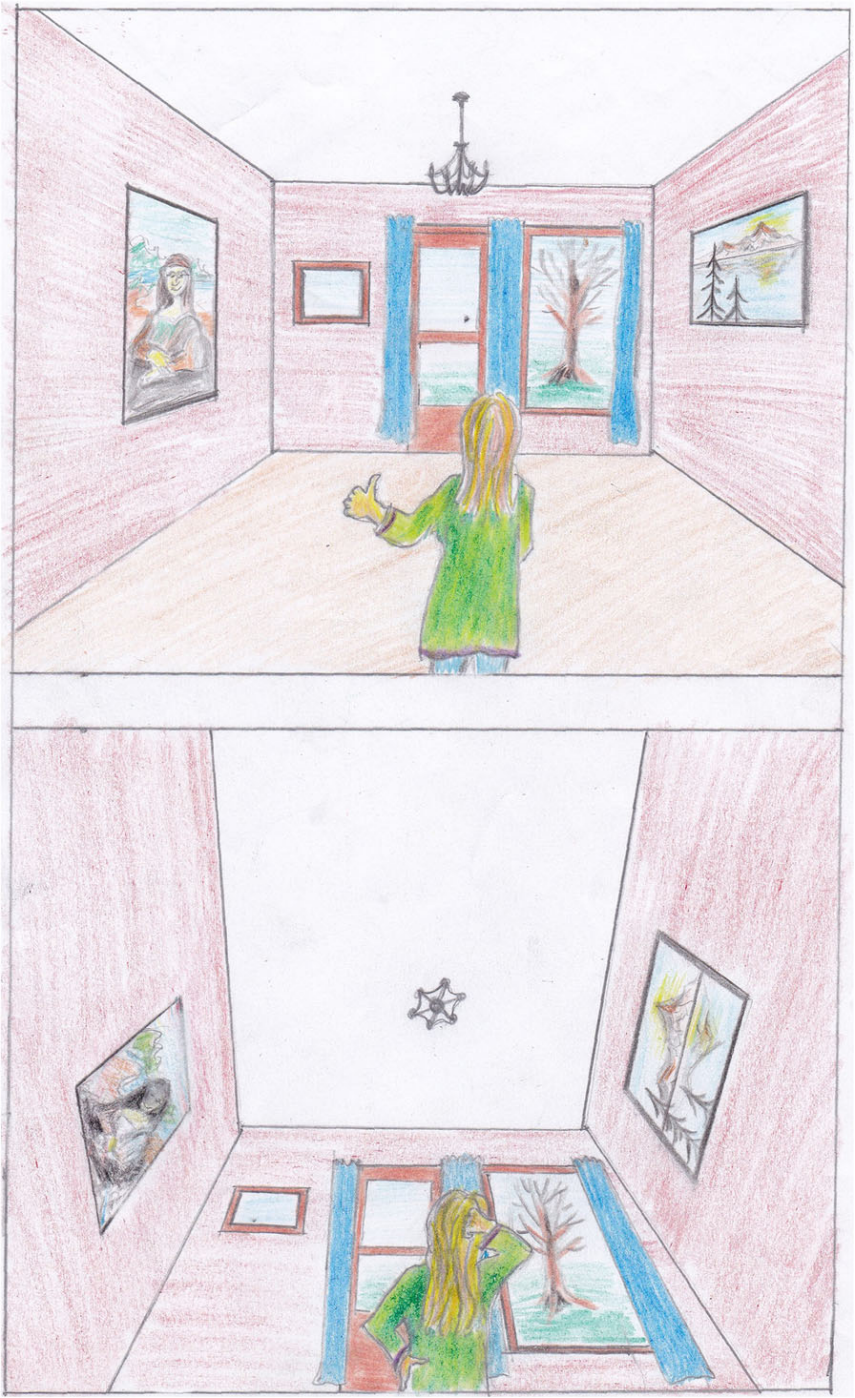
Cartoon illustrating the visual scene with normal percept and with abnormal percept in RTI with 90^o^ forward rotation of the visual scene in the sagittal plane. (Drawn by Kristoffer Arntzen)

A 63-year-old woman was admitted to the hospital due to aortoiliac occlusive disease (Fig. 1). She had a history of hypertension and chronic obstructive pulmonary disease, and was a former smoker for 50 years. Aortobifemoral graft surgery was performed without any major complications, but there was a perioperative bleeding of 1000 ml requiring blood transfusion, plasma and fluids. She was in pain, was subfebrile and was haemodynamically unstable. Norepinephrine, epidural analgesics, oxycodone tablets, antibiotics and fluids were given. In the morning on postoperative (PO) day 1 she reported dizziness, nausea, and a fluctuating “sensation that the room was turned upside-down”. It was believed that she was overdosed with opioids, and oxycodone and the epidural were discontinued. On the following day she felt much better and was mobilized, but still experienced 3 more episodes with stereotyped visual illusions, and these recurred twice on PO day 3. All episodes occurred while lying in bed. She was not able to tell whether they were triggered by turning of the head or by the use of her arms. The episodes started suddenly and faded gradually away. Each episode lasted 10 to 30 min. She described unspecific dizziness, but an exact 90^o^ rotation of the visual scene in the sagittal plane (Fig. 2). She also described a sensation of being translocated to the posterior corner of the ceiling, looking down at the room. No oscillopsia or other associated neurological symptoms were reported. The blood pressure (BP) was not measured during the episodes, and no treatment was given. After the last incident the surgeons asked for a neurological assessment.

**Fig. 3:**
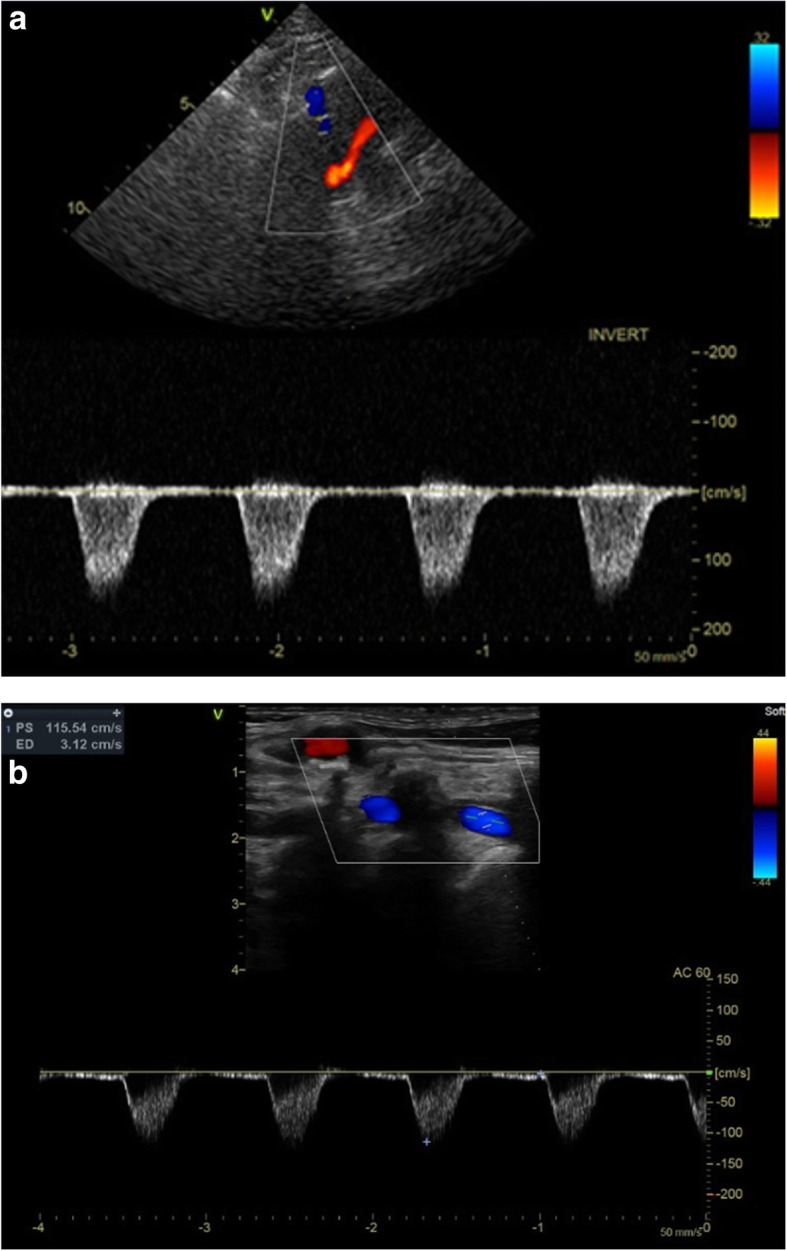
Diagnostic ultrasound using (**a**) transcranial color-coded sonography (transforaminal approach) showing retrograde flow in the left V4-VA with velocity 116 cm/s. **b** Extracranial duplex, longitudinal plane, showing complete retrograde flow in the left V2-VA with velocity 116 cm/s

**Fig. 4:**
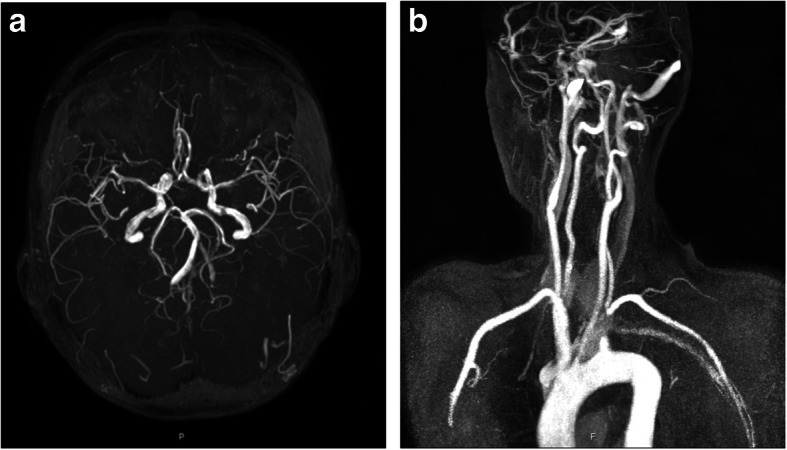
MR angiography of (**a**) cerebral arteries showing a missing left posterior communicating artery (**b**) precerebral arteries showing an occlusion of the proximal left subclavian artery

Work-up with neurological examination and head CT showed nothing amiss, but the BP on her left arm was consistently around 40 mmHg below that on the right arm. Cerebrovascular Doppler Sonography revealed complete retrograde flow from the V4 (Fig. 3a) and the V2 (Fig. 3b) segment of the left vertebral artery (VA), independent of muscular activity of the arm. Besides the standard postoperative care, no specific treatment was given. No more illusions were experienced, and the patient was discharged from hospital. Further diagnostic workup with a head magnetic resonance imaging (MRI) scan was performed 6 weeks later. This revealed an older ischemic lesion in the right external capsule, and absent flow in the left posterior communicating artery (Fig. 4a). The proximal left subclavian artery (Fig. 4b) was occluded. On follow-up 3 months after the discharge from hospital, the patient was in good condition and reporting no neurological symptoms after being discharged from the hospital. A diagnosis of subclavian steal grade 3 (permanent retrograde flow in the vertebral artery) was confirmed.

## Discussion and conclusions

When visual illusions are encountered in the clinic, diverse etiologies, which span the fields of psychiatry and neurology, must be considered carefully. The room-tilt illusion may easily be neglected or misdiagnosed if poorly known. In the present case we have described it, to our knowledge for the first time associated with subclavian steal.

Based on their review, Sierra-Hidalgo and colleagues pointed out that ischemic posterior fossa lesions, affecting the vestibular system in particular, probably have been under-recognized, and that the most common documented cause of RTI is posterior circulation stroke [2, 7]. In our patient, only an incidental ischemic brain lesion was found on the head MRI performed weeks after the episodes with RTI, but she was unquestionably at high risk of cerebral ischemia. One can only speculate if early diffusion- weighted MRI (DWI), which is sensitive for early detection of ischemic brain tissue [8], even when transient [9], could have captured an ischemic lesion in close wake of one of her RTIs. Our patient had extensive large vessel disease, and posterior cerebral ischemia was the suspected cause of her RTIs. This presumption led to a diagnosis of subclavian steal.

**Fig. 5:**
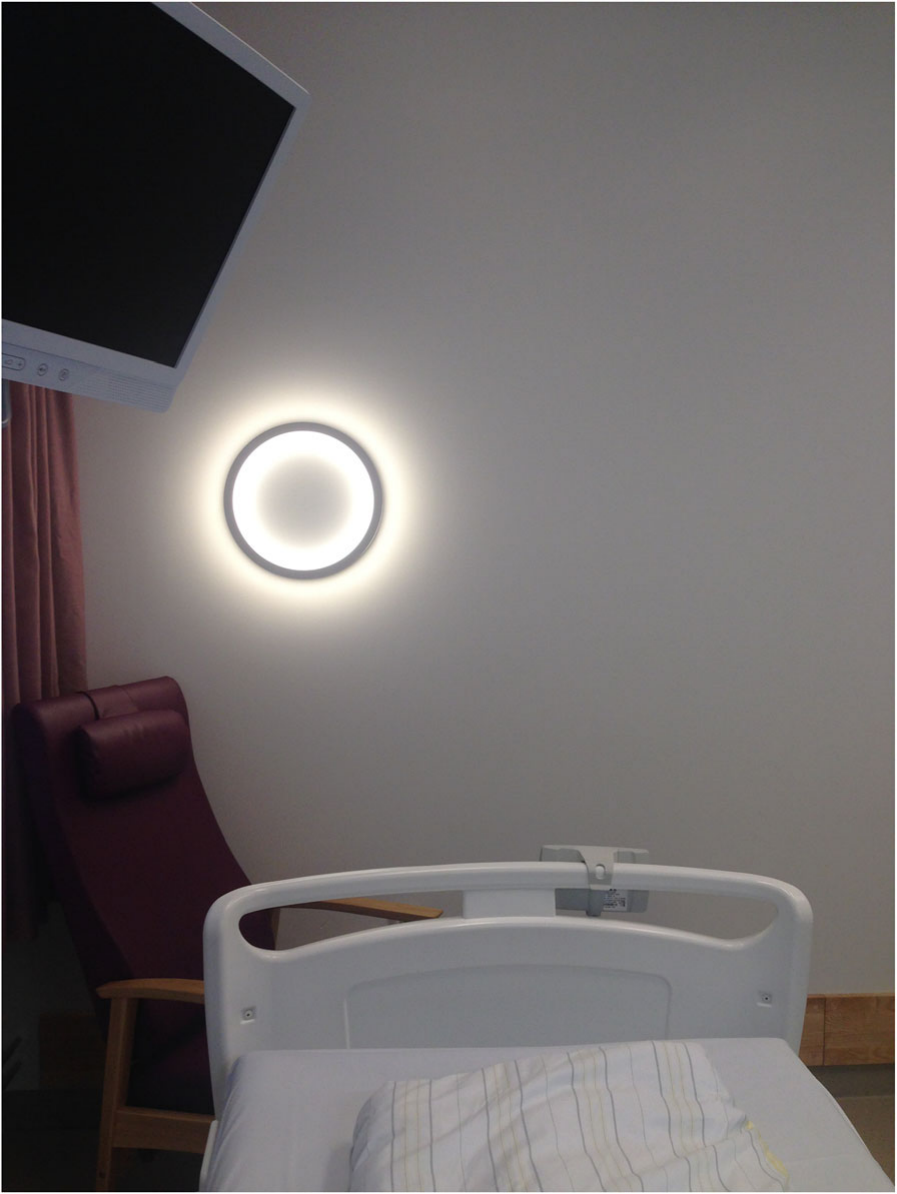
Picture of the patient’s room. Room tilt illusions are probably more likely to occur if the visual frame contains a strongly polarized object

*Subclavian steal* (SS) refers to a phenomenon of reversed flow in the vertebral artery ipsilateral to a hemodynamically significant stenosis or occlusion of the prevertebral subclavian artery [10]. In many cases, subclavian steal is asymptomatic, but when causing symptoms due to vertebro-basilar insufficiency or arterial insufficiency of the upper extremity, it becomes a syndrome. Symptoms related to ischemia of the arm are usually not prominent due to the normally slow development of the subclavian artery stenosis with the development of rich collateral supply. On the contrary, transient cerebral symptoms are common. With conventional stroke symptoms from the posterior cerebral circulation, the subclavian steal in our patient would have been considered definitively symptomatic. Still, we find it unlikely that the SS in the present case is coincidental and that alternative mechanisms caused her symptoms. Being somewhat hemodynamically unstable postoperatively probably contributed to minor transient ischemia in the posterior cerebral circulation, manifesting with dizziness and RTI. One could argue that if this was the cause, the RTIs should more likely have occurred when she was in erect rather than in supine position. On the other hand, experimental data have shown that lying horizontally and having a simple visual frame with strongly polarized objects (Fig. 5) increase the risk of forward reorientation illusions [11]. Furthermore, these are usually associated with a levitation illusion. In the present case, the patient experienced the feeling of being lifted to the ceiling during the RTIs, indicating an aberrant inner body image. River and colleagues ascribed the sense of body levitation, an alteration of the body scheme, as a result of dysfunction of the vestibular centers in the brainstem, but also noted that it could represent an epiphenomenon [12].

Traditionally, a parieto-occipital (junction) lesion was considered to be the most common cause of RTI [12], but injury to a variety of locations in both the visual-, vestibular- and the somatosensory system has been associated with the phenomenon. This is not surprising since RTI probably includes not only impairment of spatial orientation, but also spatial attention and balance control. As mentioned, the perception of self in space is based on integration of both visual, vestibular and somatosensory input [3].

The lack of detailed physical examinations during the patient’s RTIs limit the possibility to draw definite conclusions about the etiology. No otoneurologic workup was performed. Malis & Guyot reported 23 subjects with RTI that had been assessed at an otoneurologic unit over a 20-year period. All but two were found to have either a vestibular peripheral disorder or a normal assessment finding [13]. All the subjects underwent electronystagmography, and nystagmus was observed in 11. Four without nystagmus were reported to have “vestibular symptoms”, and two had positive caloric response. Thirteen subjects underwent MRI, and only one had a pathological finding. No nystagmus was observed in our patient, and she had no story of previous attacks of vertigo, hearing loss or tinnitus.

After our patient’s first RTI, delirium caused by opioid intoxication was suspected, but even 2 days after the drug discontinuation she experienced new episodes. Other possible causes of delirium such as infection and withdrawal state were also considered unlikely.

Epilepsy is mentioned as a cause of RTI in the literature, and there is some evidence that vestibular symptoms may occur as a manifestation of focal epileptic activity. To our knowledge, however, no detailed single case report describing RTI strongly associated with epilepsy has been published. No electroencephalography (EEG) was obtained from our patient.

Visual perceptual abnormalities in migraine are common, but illusion of rotation in the frontal plane, as in the present patient, is considered a very rare migraine aura symptom [6]. Our patient had no history of migraine, and experienced no headaches.

In summary, RTI is a transient disorder of the central vestibular system, associated with both peripheral and central lesions. An identifiable cause is often not found, but RTI should not be neglected. It may represent a serious underlying condition, and thorough evaluation is warranted.

## Data Availability

Clinical data about the patient is stored in her electronic medical journal at Nordland Hospital Trust.

## Abbreviations

RTI: Room tilt illusion
PO: Postoperative
BP: Blood pressure
CT: Computer tomography
VA: Vertebral artery
MRI: Magnetic resonance imaging
DWI: Diffusion weighted imaging
SS: Subclavian steal
TIA: Transient ischemic attacks
